# Supplementary material for: Smart-Plexer: a breakthrough workflow for hybrid development of multiplex PCR assays
Source: Commun Biol. 2023 Sep 9;6:922. doi: 10.1038/s42003-023-05235-w (PMC10492832; doi:10.1038/s42003-023-05235-w)
Supplement: Supplementary file 2 — Supplementary Information [file 42003_2023_5235_MOESM2_ESM.pdf]

Smart-Plexer: a breakthrough workflow for hybrid development of multiplex PCR assays

Luca Miglietta[1,2], Yuwen Chen[2,$], Zhi Luo[1,$], Ke Xu[1,2], Ning Ding[1], Tianyi Peng[2], Ahmad Moniri[2], Louis Kreitmann[1], Miguel Cacho-Soblechero[2], Alison Holmes[1], Pantelis Georgiou[2] and Jesus Rodriguez-Manzano[1*]

[1]*Department of Infectious Disease, Faculty of Medicine, Imperial College London, London, UK.*
[2]*Department of Electrical and Electronic Engineering, Faculty of Engineering, Imperial College London, London, UK.*
[$]*These authors contributed equally.*
[*]*Corresponding author: j.rodriguez-manzano@imperial.ac.uk*

Supplementary Table 1 | Fitting model performances. Data source: https://github.com/am5113/pyACA

Model name	Equation	data shape (N)	MSE
Four-parameter Sigmoid	$f(t) = \dfrac{a}{1 + exp^{-c(t-d)}} + b$	16,188	0.017085
Five-parameter Sigmoid	$f(t) = \dfrac{a}{(1 + exp^{-c(t-d)})^e} + b$	16,188	0.003603
Six-parameter Sigmoid	$f(t) = \dfrac{a}{(1 + k\, exp^{-c(t-d)})^e} + b$	16,188	0.011825

Supplementary Table 2 | Combination table for 3-plex

Multiplex assay name	HAdV singleplex	HCoV-HKU1 singleplex	MERS-CoV singleplex
PM3.01	HAdV_HEX_03	HCoV-HKU1_N_02	MERS-CoV_N_01
PM3.02	HAdV_HEX_03	HCoV-HKU1_N_02	MERS-CoV_N_03
PM3.03	HAdV_HEX_03	HCoV-HKU1_N_02	MERS-CoV_N_04
PM3.04	HAdV_HEX_03	HCoV-HKU1_N_04	MERS-CoV_N_01
PM3.05	HAdV_HEX_03	HCoV-HKU1_N_04	MERS-CoV_N_03
PM3.06	HAdV_HEX_03	HCoV-HKU1_N_04	MERS-CoV_N_04
PM3.07	HAdV_HEX_03	HCoV-HKU1_N_06	MERS-CoV_N_01
PM3.08	HAdV_HEX_03	HCoV-HKU1_N_06	MERS-CoV_N_03
PM3.09	HAdV_HEX_03	HCoV-HKU1_N_06	MERS-CoV_N_04
PM3.10	HAdV_HEX_09	HCoV-HKU1_N_02	MERS-CoV_N_01
PM3.11	HAdV_HEX_09	HCoV-HKU1_N_02	MERS-CoV_N_03
PM3.12	HAdV_HEX_09	HCoV-HKU1_N_02	MERS-CoV_N_04
PM3.13	HAdV_HEX_09	HCoV-HKU1_N_04	MERS-CoV_N_01
PM3.14	HAdV_HEX_09	HCoV-HKU1_N_04	MERS-CoV_N_03
PM3.15	HAdV_HEX_09	HCoV-HKU1_N_04	MERS-CoV_N_04
PM3.16	HAdV_HEX_09	HCoV-HKU1_N_06	MERS-CoV_N_01
PM3.17	HAdV_HEX_09	HCoV-HKU1_N_06	MERS-CoV_N_03
PM3.18	HAdV_HEX_09	HCoV-HKU1_N_06	MERS-CoV_N_04
PM3.19	HAdV_HEX_12	HCoV-HKU1_N_02	MERS-CoV_N_01
PM3.20	HAdV_HEX_12	HCoV-HKU1_N_02	MERS-CoV_N_03
PM3.21	HAdV_HEX_12	HCoV-HKU1_N_02	MERS-CoV_N_04
PM3.22	HAdV_HEX_12	HCoV-HKU1_N_04	MERS-CoV_N_01
PM3.23	HAdV_HEX_12	HCoV-HKU1_N_04	MERS-CoV_N_03
PM3.24	HAdV_HEX_12	HCoV-HKU1_N_04	MERS-CoV_N_04
PM3.25	HAdV_HEX_12	HCoV-HKU1_N_06	MERS-CoV_N_01
PM3.26	HAdV_HEX_12	HCoV-HKU1_N_06	MERS-CoV_N_03
PM3.27	HAdV_HEX_12	HCoV-HKU1_N_06	MERS-CoV_N_04

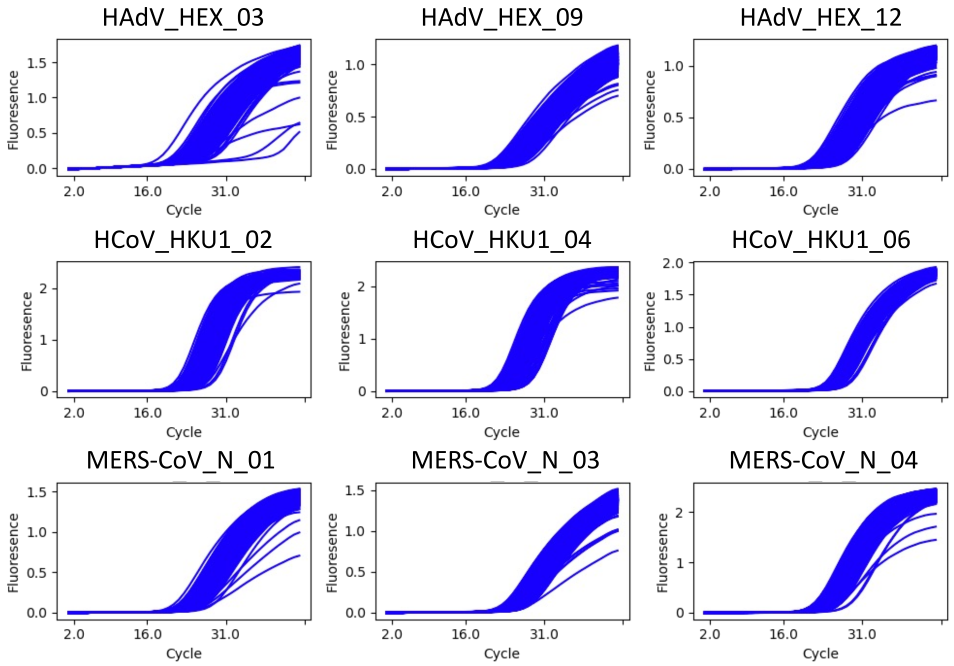

Supplementary Fig. 1 | Raw curve from Smart-Plexer validation using a 3-plex assay.

Supplementary Table 3 | Raw curve details from Smart-Plexer validation using a 3-plex assay.

Target	Assay	Curve Counts	C_t values Means	C_t STD	Minimum C_t	Maximum C_t
**Adenovirus**	**HAdV_HEX_03**	639	24.66	1.43	19.12	40.57
**Adenovirus**	**HAdV_HEX_09**	563	27.50	1.10	23.78	31.16
**Adenovirus**	**HAdV_HEX_12**	581	26.48	1.15	23.00	30.37
**Coronavirus HKU1**	**HCoV-HKU1_N_02**	606	24.39	0.99	21.39	28.82
**Coronavirus HKU1**	**HCoV-HKU1_N_04**	598	24.47	1.06	21.72	28.45
**Coronavirus HKU1**	**HCoV-HKU1_N_06**	149	26.47	0.92	24.64	29.53
**MERS**	**MERS-CoV_N_01**	666	25.49	1.02	22.51	30.95
**MERS**	**MERS-CoV_N_03**	386	27.00	0.99	24.63	30.93
**MERS**	**MERS-CoV_N_04**	744	24.07	1.00	21.89	28.96

Supplementary Table 4 | ADS and MDS scores for the three curve representations in 3-plex

		Raw curve		Normalised on FFI curve		5 fitted parameters	
Combination	Multiplex type	ADS	MDS	ADS	MDS	ADS	MDS
PM3.01	Simulated	2.544	0.585	0.434	0.130	66.089	5.489
PM3.01	Empirical	1.279	0.903	0.488	0.068	12.764	6.693
PM3.02	Simulated	2.978	1.239	0.655	0.440	66.115	5.489
PM3.02	Empirical	1.804	0.537	0.721	0.443	6.999	1.378
PM3.03	Simulated	2.260	0.647	0.393	0.261	8.067	5.489
PM3.03	Empirical	2.021	1.008	0.438	0.128	5.599	2.232
PM3.04	Simulated	2.462	0.585	0.401	0.130	65.369	4.357
PM3.04	Empirical	1.848	1.198	0.396	0.129	15.182	5.940
PM3.05	Simulated	2.896	1.239	0.622	0.440	65.395	4.357
PM3.05	Empirical	1.828	0.382	0.644	0.487	7.165	3.297
PM3.06	Simulated	2.174	0.514	0.359	0.261	7.290	4.357
PM3.06	Empirical	2.586	0.898	0.320	0.118	3.336	1.905
PM3.07	Simulated	0.884	0.585	0.209	0.130	64.450	4.770
PM3.07	Empirical	1.258	0.314	0.190	0.155	15.637	4.916
PM3.08	Simulated	1.286	0.810	0.334	0.265	64.535	4.989
PM3.08	Empirical	0.654	0.495	0.397	0.178	6.503	2.948
PM3.09	Simulated	1.999	0.810	0.321	0.261	62.730	7.012
PM3.09	Empirical	3.197	1.822	0.263	0.171	7.041	1.976
PM3.10	Simulated	3.638	1.664	0.644	0.378	66.659	2.998
PM3.10	Empirical	1.925	0.867	0.769	0.266	67.390	2.001
PM3.11	Simulated	3.654	1.066	0.656	0.059	66.601	2.779
PM3.11	Empirical	1.777	0.619	0.789	0.129	67.853	3.792
PM3.12	Simulated	3.734	0.647	0.646	0.338	66.257	11.699
PM3.12	Empirical	2.674	0.966	0.749	0.561	66.970	10.969
PM3.13	Simulated	3.558	1.664	0.611	0.378	65.969	2.998
PM3.13	Empirical	2.494	0.751	0.692	0.272	66.407	1.132
PM3.14	Simulated	3.574	1.066	0.622	0.059	65.910	2.779
PM3.14	Empirical	1.461	1.141	0.787	0.131	67.556	2.493
PM3.15	Simulated	3.650	0.514	0.613	0.287	65.509	10.501
PM3.15	Empirical	2.645	1.799	0.728	0.530	67.005	17.366
PM3.16	Simulated	1.930	1.258	0.292	0.199	5.179	2.998
PM3.16	Empirical	2.228	0.548	0.307	0.214	5.590	1.088
PM3.17	Simulated	1.914	1.066	0.208	0.059	5.178	2.779
PM3.17	Empirical	2.084	0.615	0.234	0.092	6.334	3.364
PM3.18	Simulated	3.425	2.304	0.448	0.301	61.079	7.767
PM3.18	Empirical	3.026	1.571	0.401	0.304	64.531	9.985
PM3.19	Simulated	3.207	1.027	0.422	0.091	65.674	2.055
PM3.19	Empirical	3.176	2.246	0.485	0.134	65.996	1.461
PM3.20	Simulated	3.261	0.542	0.636	0.382	65.703	2.055
PM3.20	Empirical	2.532	1.213	0.781	0.525	66.492	1.275
PM3.21	Simulated	3.301	0.647	0.392	0.259	7.947	2.055
PM3.21	Empirical	3.375	0.757	0.399	0.117	5.731	1.125
PM3.22	Simulated	3.127	1.027	0.388	0.091	65.057	1.230
PM3.22	Empirical	3.071	1.789	0.436	0.172	66.130	1.302
PM3.23	Simulated	3.181	0.542	0.603	0.382	65.085	1.230
PM3.23	Empirical	1.857	0.940	0.761	0.488	66.533	1.742
PM3.24	Simulated	3.217	0.514	0.359	0.259	7.272	1.230
PM3.24	Empirical	3.252	0.479	0.292	0.168	4.574	1.999
PM3.25	Simulated	1.511	1.027	0.149	0.091	65.860	4.770
PM3.25	Empirical	3.396	2.002	0.145	0.092	65.538	6.607
PM3.26	Simulated	1.534	0.542	0.268	0.157	65.947	4.989
PM3.26	Empirical	1.799	1.349	0.370	0.129	67.646	7.135
PM3.27	Simulated	3.004	2.249	0.273	0.157	64.435	10.085
PM3.27	Empirical	2.027	1.391	0.274	0.097	65.362	1.782

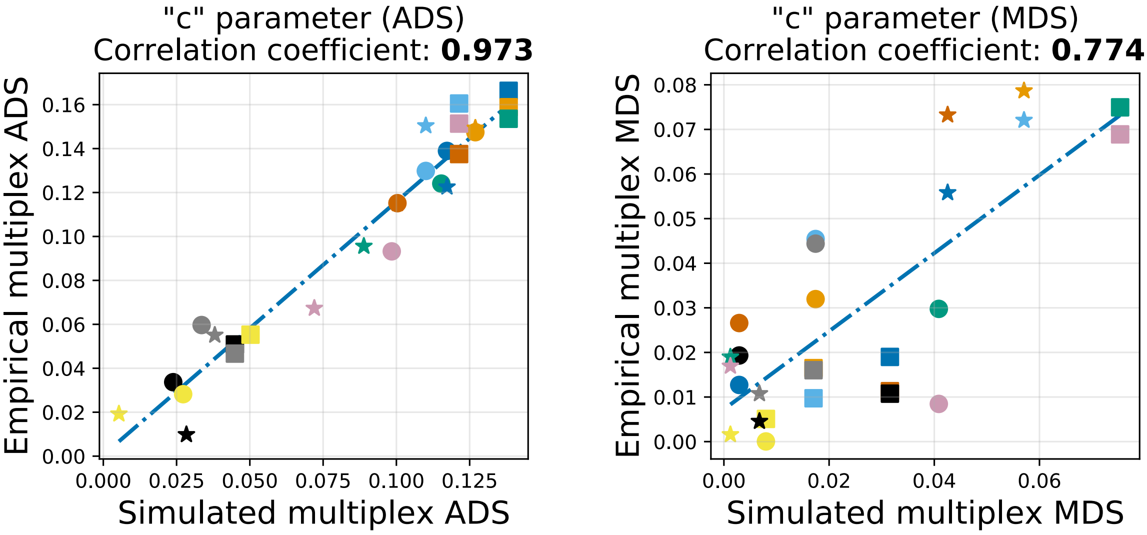

Supplementary Fig. 2 | Correlation of "c" ADS and MDS for 3-plex.

Supplementary Table 5 | "c" parameter stats for 3-plex

Multiplex assay name	Simulated ADS	Empirical ADS	Simulated MDS	Empirical MDS	RMSE	Mean Silouette Score (MMS)	Cross-validation ACA accuracy
PM3.01	0.117	0.139	0.003	0.013	0.022	0.218	98.97
PM3.02	0.127	0.147	0.017	0.032	0.018	0.365	98.86
PM3.03	0.115	0.124	0.041	0.030	0.018	0.264	99.90
PM3.04	0.100	0.115	0.003	0.027	0.018	0.291	99.55
PM3.05	0.110	0.130	0.017	0.045	0.022	0.399	98.92
PM3.06	0.098	0.093	0.041	0.008	0.024	0.306	99.66
PM3.07	0.024	0.034	0.003	0.019	0.020	0.249	99.28
PM3.08	0.034	0.060	0.017	0.044	0.028	0.342	98.86
PM3.09	0.027	0.028	0.008	0.001	0.009	0.241	99.82
PM3.10	0.138	0.166	0.032	0.019	0.041	0.310	99.75
PM3.11	0.138	0.159	0.017	0.016	0.027	0.210	99.35
PM3.12	0.138	0.154	0.075	0.075	0.019	0.376	99.90
PM3.13	0.121	0.138	0.032	0.011	0.031	0.274	99.75
PM3.14	0.121	0.161	0.017	0.010	0.050	0.122	99.88
PM3.15	0.121	0.151	0.075	0.069	0.040	0.485	99.90
PM3.16	0.045	0.051	0.032	0.011	0.021	0.365	99.50
PM3.17	0.045	0.047	0.017	0.016	0.003	0.211	98.63
PM3.18	0.050	0.055	0.008	0.005	0.008	0.282	99.04
PM3.19	0.117	0.123	0.043	0.056	0.009	0.391	99.95
PM3.20	0.127	0.149	0.057	0.079	0.025	0.396	99.64
PM3.21	0.089	0.096	0.001	0.019	0.014	0.152	99.75
PM3.22	0.100	0.115	0.043	0.073	0.022	0.452	100.00
PM3.23	0.110	0.150	0.057	0.072	0.044	0.414	99.52
PM3.24	0.072	0.067	0.001	0.017	0.017	0.220	99.74
PM3.25	0.028	0.010	0.007	0.005	0.027	0.093	99.88
PM3.26	0.038	0.055	0.007	0.011	0.020	0.253	99.29
PM3.27	0.005	0.019	0.001	0.002	0.019	0.147	99.51

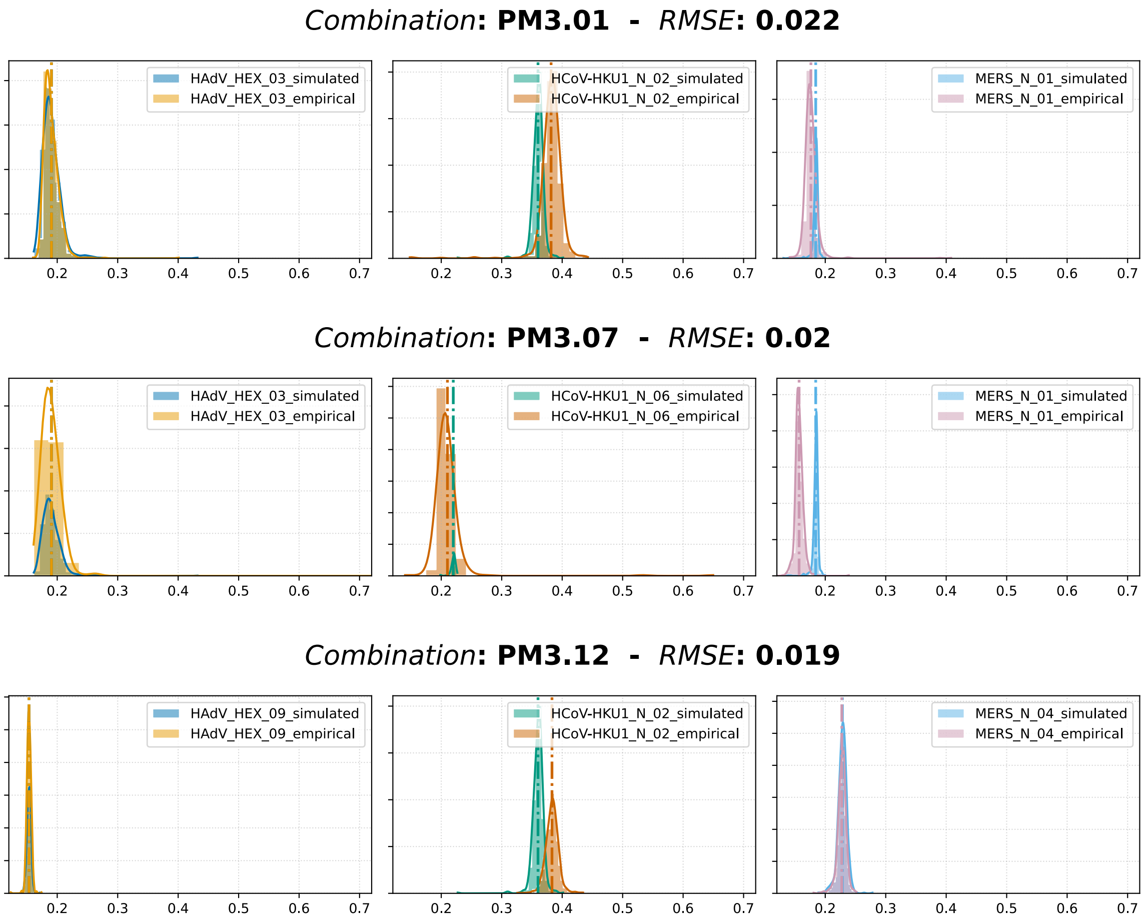

Supplementary Fig. 3. | Distribution curves of simulated multiplex and empirical multiplex for each target. The figure displays the distribution “c” of the simulated and empirical multiplex for each target. The x-axis represents the parameter values, while the y-axis indicates the frequency.

Supplementary Table 6| Combination table for 7-plex

Assay Name	HAdVnovirus Singleplex	Coronavirus 229E Singleplex	Coronavirus HKU1 Singleplex	Coronavirus NL63 Singleplex	Coronavirus OC43 Singleplex	SARS-CoV-2 Singleplex	MERS Singleplex
PM7.1176	HAdV_HEX_06	HCoV-229E_N_01	HCoV-HKU1_N_02	HCoV-NL63_N_01	HCoV-OC43_N_02	SARS-CoV-2_N_03	MERS_N_04
PM7.1191	HAdV_HEX_06	HCoV-229E_N_01	HCoV-HKU1_N_02	HCoV-NL63_N_02	HCoV-OC43_N_01	SARS-CoV-2_N_01	MERS_N_03
PM7.1286	HAdV_HEX_06	HCoV-229E_N_01	HCoV-HKU1_N_02	HCoV-NL63_N_04	HCoV-OC43_N_04	SARS-CoV-2_N_01	MERS_N_02
PM7.1294	HAdV_HEX_06	HCoV-229E_N_01	HCoV-HKU1_N_02	HCoV-NL63_N_04	HCoV-OC43_N_04	SARS-CoV-2_N_03	MERS_N_02
PM7.1318	HAdV_HEX_06	HCoV-229E_N_01	HCoV-HKU1_N_04	HCoV-NL63_N_01	HCoV-OC43_N_02	SARS-CoV-2_N_03	MERS_N_02
PM7.1319	HAdV_HEX_06	HCoV-229E_N_01	HCoV-HKU1_N_04	HCoV-NL63_N_01	HCoV-OC43_N_02	SARS-CoV-2_N_03	MERS_N_03
PM7.1339	HAdV_HEX_06	HCoV-229E_N_01	HCoV-HKU1_N_04	HCoV-NL63_N_02	HCoV-OC43_N_01	SARS-CoV-2_N_02	MERS_N_03
PM7.1430	HAdV_HEX_06	HCoV-229E_N_01	HCoV-HKU1_N_04	HCoV-NL63_N_04	HCoV-OC43_N_04	SARS-CoV-2_N_01	MERS_N_02
PM7.1449	HAdV_HEX_06	HCoV-229E_N_01	HCoV-HKU1_N_06	HCoV-NL63_N_01	HCoV-OC43_N_01	SARS-CoV-2_N_03	MERS_N_01
PM7.1451	HAdV_HEX_06	HCoV-229E_N_01	HCoV-HKU1_N_06	HCoV-NL63_N_01	HCoV-OC43_N_01	SARS-CoV-2_N_03	MERS_N_03
PM7.1593	HAdV_HEX_06	HCoV-229E_N_01	HCoV-HKU1_N_08	HCoV-NL63_N_01	HCoV-OC43_N_01	SARS-CoV-2_N_03	MERS_N_01
PM7.1595	HAdV_HEX_06	HCoV-229E_N_01	HCoV-HKU1_N_08	HCoV-NL63_N_01	HCoV-OC43_N_01	SARS-CoV-2_N_03	MERS_N_03
PM7.2014	HAdV_HEX_06	HCoV-229E_N_02	HCoV-HKU1_N_04	HCoV-NL63_N_04	HCoV-OC43_N_04	SARS-CoV-2_N_03	MERS_N_02
PM7.2151	HAdV_HEX_06	HCoV-229E_N_02	HCoV-HKU1_N_06	HCoV-NL63_N_04	HCoV-OC43_N_04	SARS-CoV-2_N_01	MERS_N_03
PM7.2155	HAdV_HEX_06	HCoV-229E_N_02	HCoV-HKU1_N_06	HCoV-NL63_N_04	HCoV-OC43_N_04	SARS-CoV-2_N_02	MERS_N_03
PM7.2203	HAdV_HEX_06	HCoV-229E_N_02	HCoV-HKU1_N_08	HCoV-NL63_N_02	HCoV-OC43_N_01	SARS-CoV-2_N_02	MERS_N_03
PM7.2295	HAdV_HEX_06	HCoV-229E_N_02	HCoV-HKU1_N_08	HCoV-NL63_N_04	HCoV-OC43_N_04	SARS-CoV-2_N_01	MERS_N_03
PM7.2302	HAdV_HEX_06	HCoV-229E_N_02	HCoV-HKU1_N_08	HCoV-NL63_N_04	HCoV-OC43_N_04	SARS-CoV-2_N_03	MERS_N_02
PM7.2303	HAdV_HEX_06	HCoV-229E_N_02	HCoV-HKU1_N_08	HCoV-NL63_N_04	HCoV-OC43_N_04	SARS-CoV-2_N_03	MERS_N_03
PM7.2601	HAdV_HEX_09	HCoV-229E_N_01	HCoV-HKU1_N_06	HCoV-NL63_N_01	HCoV-OC43_N_01	SARS-CoV-2_N_03	MERS_N_01
PM7.2602	HAdV_HEX_09	HCoV-229E_N_01	HCoV-HKU1_N_06	HCoV-NL63_N_01	HCoV-OC43_N_01	SARS-CoV-2_N_03	MERS_N_02
PM7.4382	HAdV_HEX_12	HCoV-229E_N_02	HCoV-HKU1_N_06	HCoV-NL63_N_02	HCoV-OC43_N_04	SARS-CoV-2_N_01	MERS_N_02
PM7.4441	HAdV_HEX_12	HCoV-229E_N_02	HCoV-HKU1_N_06	HCoV-NL63_N_04	HCoV-OC43_N_02	SARS-CoV-2_N_01	MERS_N_01
PM7.4443	HAdV_HEX_12	HCoV-229E_N_02	HCoV-HKU1_N_06	HCoV-NL63_N_04	HCoV-OC43_N_02	SARS-CoV-2_N_01	MERS_N_03

Supplementary Table 7 | “c” parameter stats 7-plex

Multiplex assay name	Simulated ADS	Empirical ADS	Simulated MDS	Empirical MDS	RMSE	Mean Silouette Score (MMS)	Cross-validation ACA accuracy
PM7.1176	0.098	0.101	0.012	0.002	0.011	0.382	59.44%
PM7.1191	0.098	0.107	0.013	0.018	0.009	0.441	76.54%
PM7.1286	0.190	0.188	0.024	0.003	0.026	0.345	82.88%
PM7.1294	0.193	0.189	0.024	0.006	0.019	0.379	91.99%
PM7.1318	0.098	0.092	0.012	0.002	0.029	0.381	60.38%
PM7.1319	0.097	0.090	0.012	0.002	0.031	0.521	83.45%
PM7.1339	0.097	0.089	0.013	0.004	0.018	0.426	94.01%
PM7.1430	0.191	0.184	0.024	0.010	0.026	0.330	71.62%
PM7.1449	0.034	0.039	0.000	0.004	0.012	0.591	93.64%
PM7.1451	0.035	0.041	0.000	0.001	0.014	0.557	95.32%
PM7.1593	0.034	0.041	0.000	0.001	0.012	0.555	92.92%
PM7.1595	0.035	0.041	0.000	0.003	0.010	0.393	94.41%
PM7.2014	0.195	0.206	0.007	0.007	0.035	0.576	90.46%
PM7.2151	0.190	0.182	0.037	0.012	0.031	0.456	97.10%
PM7.2155	0.190	0.182	0.037	0.016	0.023	0.484	70.63%
PM7.2203	0.098	0.120	0.012	0.008	0.033	0.520	92.71%
PM7.2295	0.192	0.180	0.025	0.009	0.036	0.574	95.51%
PM7.2302	0.195	0.208	0.002	0.005	0.024	0.561	88.85%
PM7.2303	0.192	0.191	0.002	0.006	0.041	0.538	84.60%
PM7.2601	0.028	0.031	0.000	0.001	0.006	0.626	98.86%
PM7.2602	0.032	0.034	0.000	0.001	0.010	0.527	97.34%
PM7.4382	0.155	0.188	0.023	0.033	0.033	0.691	97.16%
PM7.4441	0.145	0.157	0.023	0.018	0.026	0.612	91.72%
PM7.4443	0.146	0.163	0.023	0.022	0.031	0.424	85.25%

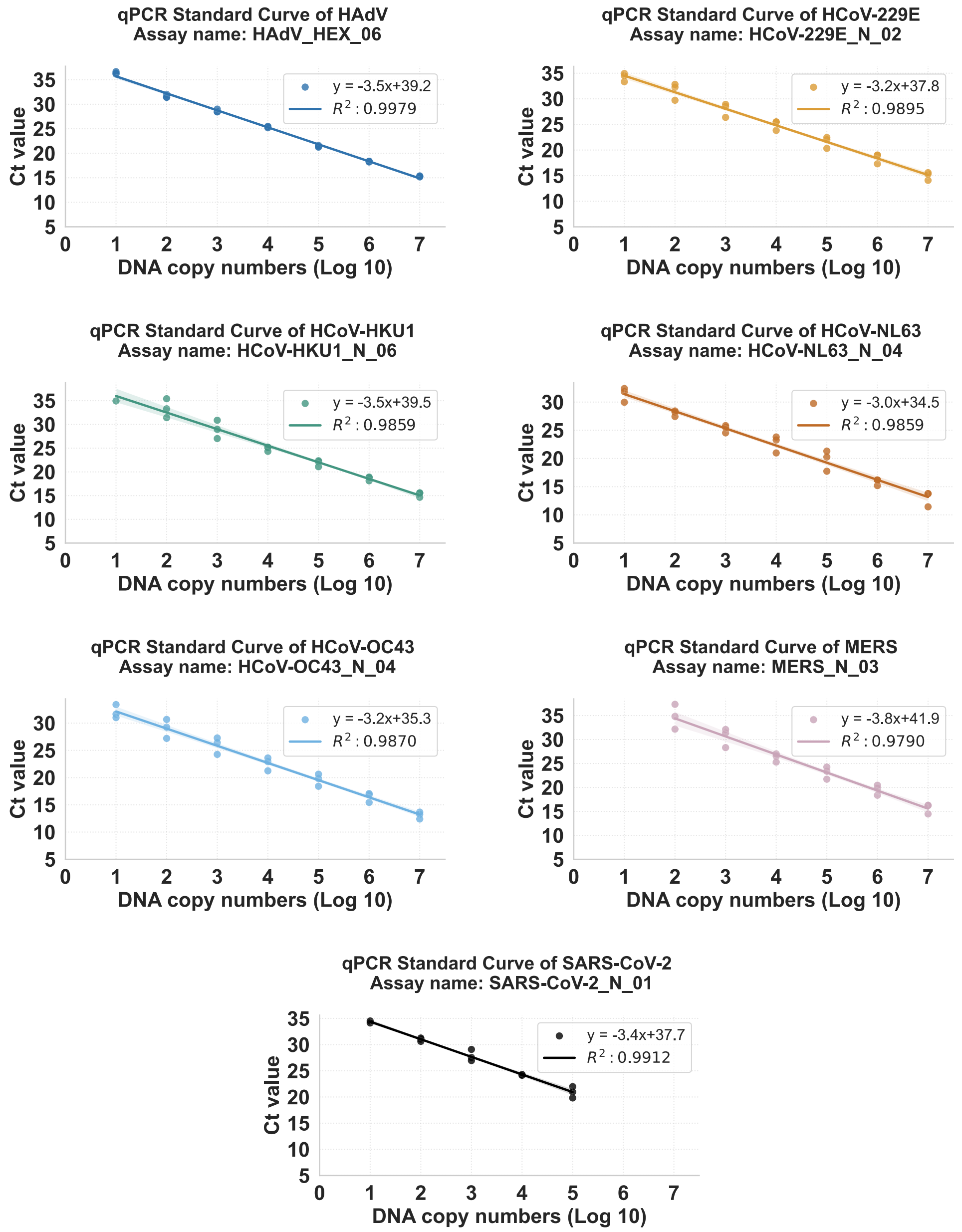

Supplementary Fig. 4 | Standard curves for all targets in the BEST selected 7-plex.

Supplementary Table 8 | Primer table for 3-plex

Oligo name	Target name	Oligo type	Oligo modification	Oligo sequence	Tm (℃)	GC cont (%)	Length (nt)	A	C	G	T	hairpin Tm (℃)	hairpin dg (kcal/mol)	PD dg (kcal/mol)	MW (g/mol)
**HAdV_01**	HEX	forward		CCCTTCGATGATGCCGCA	65.98	61.11	18	3	7	4	4	0.00	0.00	-3.21	5515.50
**HAdV_02**	HEX	forward		CGCAGTGGTCTTACATGCACATCTC	69.72	52.00	25	5	8	5	7	33.15	0.29	-3.28	7672.88
**HAdV_03**	HEX	probe	56-FAM / Z	CCTCGGAGTACCTRAGCCCCGG	72.94	70.45	22	3.5	9	6.5	3	34.87	0.20	-2.51	6769.29
**HAdV_04**	HEX	probe	56-FAM / Z	CCGCGCCACCGAGACGTACTTCAG	71.78	66.67	24	5	10	6	3	32.39	0.32	-4.89	7363.68
**HAdV_05**	HEX	reverse		CAGGCTGAAGTACGTCTCGGT	67.57	57.14	21	4	5	7	5	0.00	0.00	-5.80	6542.16
**HAdV_07**	HEX	reverse		CGCAGCGTCAAACGCTG	65.38	64.71	17	4	6	5	2	64.49	-2.57	-9.64	5260.35
**HCoV-HKU1_02**	N	forward		TCAAGAAGCTATCCCTACTAGGT	63.71	43.48	23	7	6	4	6	27.11	0.55	-2.01	7087.53
**HCoV-HKU1_03**	N	probe	56-FAM / Z	CGCCTGGTACGATTTTGCCTCAAGGCT	72.21	55.56	27	4	8	7	8	56.20	-1.74	-6.13	8322.28
**HCoV-HKU1_05**	N	reverse		AGACCTTCCTGAGCCTTCAACA	67.43	50.00	22	6	8	3	5	27.54	0.61	-1.73	6719.29
**HCoV-HKU1_06**	N	reverse		CTATTAGAAGCAGACCTTCCTGA	63.02	43.48	23	7	6	4	6	44.73	-0.74	-3.13	7087.53
**HCoV-HKU1_08**	N	reverse		GCGATCTCATCAGCCATATCAGGT	68.29	50.00	24	6	7	5	6	0.00	0.00	-3.15	7392.72
**MERS-CoV_01**	N	forward		ACGCGGAACCCTAACAATGATT	66.51	45.45	22	8	6	4	4	0.00	0.00	-6.41	6792.35
**MERS-CoV_02**	N	probe	56-FAM / Z	TGCCTCCAGTCCCCTCAATGTGGA	70.33	58.33	24	4	9	5	6	30.16	0.62	-3.02	7344.67
**MERS-CoV_03**	N	reverse		GCTAGAGGCTCTTGAAGATGATTGA	66.30	44.00	25	7	3	8	7	36.12	0.06	-7.10	7841.01
**MERS-CoV_04**	N	forward		CCACAAGCGCACTTCCACCAA	69.80	57.14	21	7	10	2	2	27.31	0.52	-6.40	6369.08
**MERS-CoV_05**	N	probe	56-FAM / Z	TTCCCTGGAGGTCTCCTGGTCCGC	72.33	66.67	24	1	9	7	7	51.20	-1.89	-6.78	7367.65
**MERS-CoV_06**	N	reverse		GTGGGTCCTCAGTGCCGAGT	69.98	65.00	20	2	5	8	5	0.00	0.00	-1.99	6244.95

Supplementary Table 9 | Assay table for 3-plex

Assay_ID	Forward_ID	Probe_ID	Reverse_ID
**HAdV_HEX_03**	HAdV_01	HAdV_04	HAdV_05
**HAdV_HEX_09**	HAdV_01	HAdV_03	HAdV_07
**HAdV_HEX_12**	HAdV_02	HAdV_04	HAdV_07
**HCoV-HKU1_N_02**	HCoV-HKU1_02	HCoV-HKU1_03	HCoV-HKU1_05
**HCoV-HKU1_N_04**	HCoV-HKU1_02	HCoV-HKU1_03	HCoV-HKU1_06
**HCoV-HKU1_N_06**	HCoV-HKU1_02	HCoV-HKU1_03	HCoV-HKU1_08
**MERS-CoV_N_01**	MERS-CoV_01	MERS-CoV_02	MERS-CoV_03
**MERS-CoV_N_03**	MERS-CoV_01	MERS-CoV_05	MERS-CoV_06
**MERS-CoV_N_04**	MERS-CoV_04	MERS-CoV_05	MERS-CoV_06

Supplementary Table 10 | Primer table for 7-plex

Oligo name	Target name	Oligo type	Oligo modification	Oligo sequence	Tm (℃)	GC cont (%)	Length (nt)	A	C	G	T	hairpin Tm (℃)	hairpin dg (kcal/mol)	PD dg (kcal/mol)	MW (g/mol)
**HAdV_01**	HEX	forward		CCCTTCGATGATGCCGCA	65.98	61.11	18	3	7	4	4	0.00	0.00	-3.21	5515.50
**HAdV_02**	HEX	forward		CGCAGTGGTCTTACATGCACATCTC	69.72	52.00	25	5	8	5	7	33.15	0.29	-3.28	7672.88
**HAdV_03**	HEX	probe	56-FAM /	CCTCGGAGTACCTRAGCCCCGG	72.94	70.45	22	3.5	9	6.5	3	34.87	0.20	-2.51	6769.29
**HAdV_04**	HEX	probe	56-FAM /	CCGCGCCACCGAGACGTACTTCAG	71.78	66.67	24	5	10	6	3	32.39	0.32	-4.89	7363.68
**HAdV_06**	HEX	reverse		GCCACCGTGGGGTTTCTAAACTTG	70.15	54.17	24	4	6	7	7	41.53	-0.22	-6.02	7439.73
**HAdV_07**	HEX	reverse		CGCAGCGTCAAACGCTG	65.38	64.71	17	4	6	5	2	64.49	-2.57	-9.64	5260.35
**HCoV-229E_01**	N	forward		CAGTCAAATGGGCTGATGCA	64.66	50.00	20	6	4	6	4	39.73	-0.17	-3.12	6245.99
**HCoV-229E_02**	N	probe	56-FAM /	ACCCTGACGACCACGTTGTGGTTCA	71.10	56.00	25	5	8	6	6	55.56	-1.59	-5.96	7697.90
**HCoV-229E_03**	N	reverse		TTGTTCACTATCAACAAGCAAAGG	63.87	37.50	24	9	5	4	6	33.18	0.39	-1.78	7424.77
**HCoV-229E_04**	N	forward		GAAATGCAAAAGCCACGGTGGAA	68.67	47.83	23	10	4	7	2	35.79	0.06	-8.73	7219.64
**HCoV-229E_05**	N	probe	56-FAM /	AGTTGTGGTCAAGGTCTCTGGGGCC	71.21	60.00	25	3	5	10	7	30.39	0.49	-3.93	7824.96
**HCoV-229E_06**	N	reverse		AGCTCAGCAAATTGTGGATAGCC	67.37	47.83	23	7	5	6	5	0.00	0.00	-4.10	7152.57
**HCoV-HKU1_02**	N	forward		TCAAGAAGCTATCCCTACTAGGT	63.71	43.48	23	7	6	4	6	27.11	0.55	-2.01	7087.53
**HCoV-HKU1_03**	N	probe	56-FAM /	CGCCTGGTACGATTTTGCCTCAAGGCT	72.21	55.56	27	4	8	7	8	56.20	-1.74	-6.13	8322.28
**HCoV-HKU1_05**	N	reverse		AGACCTTCCTGAGCCTTCAACA	67.43	50.00	22	6	8	3	5	27.54	0.61	-1.73	6719.29
**HCoV-HKU1_06**	N	reverse		CTATTAGAAGCAGACCTTCCTGA	63.02	43.48	23	7	6	4	6	44.73	-0.74	-3.13	7087.53
**HCoV-HKU1_07**	N	probe	56-FAM /	ACG[+T]TCTC[+A]ATCA[+C]GTGG[+A]CCCA	66.78	52.17	23	6	8	4	5	42.70	-0.50	-6.40	7048.50
**HCoV-HKU1_08**	N	reverse		GCGATCTCATCAGCCATATCAGGT	68.29	50.00	24	6	7	5	6	0.00	0.00	-3.15	7392.72
**HCoV-NL63_01**	N	forward		TGGTTAGTTCTGATAAGGCACC	63.67	45.45	22	5	4	6	7	0.00	0.00	-2.65	6845.36
**HCoV-NL63_02**	N	probe	56-FAM /	TGGAATGTTCAAGAGCGTTGGCGTATGCG	72.59	51.72	29	6	4	11	8	44.09	-0.44	-3.35	9108.79
**HCoV-NL63_03**	N	reverse		GGAGGCAAATCAACACGTTG	63.48	50.00	20	7	4	6	3	40.08	-0.26	-2.67	6255.00
**HCoV-NL63_04**	N	forward		GGTGCTAAAACTGTTAATACCAGT	62.95	37.50	24	8	4	5	7	41.35	-0.35	-2.43	7455.78
**HCoV-NL63_05**	N	probe	56-FAM /	AGGTTTCTGATTACGTTTGCGATTACCA	66.42	39.29	28	6	5	6	11	40.11	-0.23	-4.00	8664.52
**HCoV-NL63_06**	N	reverse		GCAATAGAGAACTTTGGTTCCA	62.40	40.91	22	7	4	5	6	45.46	-0.90	-1.81	6838.38
**HCoV-OC43_01**	N	forward		CTTGGTTCTCTGGAATTACTCA	61.29	40.91	22	4	5	4	9	30.32	0.53	-1.58	6771.31
**HCoV-OC43_02**	N	probe	56-FAM /	AGAAGGACAAGGTGTGCCTATTGCACCA	71.33	50.00	28	9	6	8	5	59.69	-2.26	-4.32	8726.58
**HCoV-OC43_03**	N	reverse		GTTCCCAGATAGTAAAAATACCAT	60.22	33.33	24	10	5	3	6	0.00	0.00	-0.94	7408.77
**HCoV-OC43_04**	N	forward		GGTGGAGAAATGTTAAAACTTGGAACT	66.26	37.04	27	10	2	8	7	0.00	0.00	-1.09	8491.44
**HCoV-OC43_06**	N	probe	56-FAM /	TCCCCATTCTTGCAGAACTCGCACCCA	73.13	55.56	27	6	12	3	6	35.29	0.11	-0.47	8180.21
**HCoV-OC43_07**	N	reverse		CCAAAGAAAAACGCACCAGCTG	66.94	50.00	22	10	7	4	1	36.75	0.01	-5.03	6795.37
**SARS-CoV-2_01**	N	forward		ATAATGGACCCCAAAATCAGCGA	66.41	43.48	23	10	6	4	3	32.19	0.27	-3.15	7114.57
**SARS-CoV-2_02**	N	probe	56-FAM /	CACCCCGCATTACGTTTGGTGGACC	70.86	60.00	25	4	9	6	6	39.70	-0.42	-2.49	7673.87
**SARS-CoV-2_03**	N	reverse		TCTGGTTACTGCCAGTTGAATCTG	66.66	45.83	24	4	5	6	9	47.08	-1.08	-4.28	7429.72
**SARS-CoV-2_04**	N	forward		CTGATTACAAACATTGGCCGCA	65.80	45.45	22	7	6	4	5	31.86	0.31	-4.08	6783.34
**SARS-CoV-2_05**	N	probe	56-FAM /	TGCACAATTTGCCCCCAGCGCTTCAG	73.33	57.69	26	5	10	5	6	48.48	-0.80	-6.06	7947.05
**SARS-CoV-2_06**	N	reverse		ATGCGCGACATTCCGAAGAA	66.42	50.00	20	7	5	5	3	28.52	0.39	-6.86	6214.98
**SARS-CoV-2_12**	N	forward		GACCCCAAAATCAGCGAAAT	62.45	45.00	20	9	6	3	2	0.00	0.00	-3.15	6167.97
**SARS-CoV-2_13**	N	probe	56-FAM /	ACCCCGCATTACGTTTGGTGGACC	70.17	58.33	24	4	8	6	6	38.87	-0.10	-2.49	7384.69
**SARS-CoV-2_14**	N	reverse		TCTGGTTACTGCCAGTTGAATCTG	66.66	45.83	24	4	5	6	9	47.08	-1.08	-4.28	7429.72
**MERS-CoV_01**	N	forward		ACGCGGAACCCTAACAATGATT	66.51	45.45	22	8	6	4	4	0.00	0.00	-6.41	6792.35
**MERS-CoV_02**	N	probe	56-FAM /	TGCCTCCAGTCCCCTCAATGTGGA	70.33	58.33	24	4	9	5	6	30.16	0.62	-3.02	7344.67
**MERS-CoV_03**	N	reverse		GCTAGAGGCTCTTGAAGATGATTGA	66.30	44.00	25	7	3	8	7	36.12	0.06	-7.10	7841.01
**MERS-CoV_04**	N	forward		CCACAAGCGCACTTCCACCAA	69.80	57.14	21	7	10	2	2	27.31	0.52	-6.40	6369.08
**MERS-CoV_05**	N	probe	56-FAM /	TTCCCTGGAGGTCTCCTGGTCCGC	72.33	66.67	24	1	9	7	7	51.20	-1.89	-6.78	7367.65
**MERS-CoV_06**	N	reverse		GTGGGTCCTCAGTGCCGAGT	69.98	65.00	20	2	5	8	5	0.00	0.00	-1.99	6244.95

Supplementary Table 11 | Assay table for 7-plex

Assay_ID	Forward_ID	Probe_ID	Reverse_ID
HAdV_HEX_06	HAdV_02	HAdV_03	HAdV_06
HAdV_HEX_09	HAdV_01	HAdV_03	HAdV_07
HAdV_HEX_12	HAdV_02	HAdV_04	HAdV_07
HCoV-229E_N_01	HCoV-229E_01	HCoV-229E_02	HCoV-229E_03
HCoV-229E_N_02	HCoV-229E_04	HCoV-229E_05	HCoV-229E_06
HCoV-HKU1_N_02	HCoV-HKU1_02	HCoV-HKU1_03	HCoV-HKU1_05
HCoV-HKU1_N_04	HCoV-HKU1_02	HCoV-HKU1_03	HCoV-HKU1_06
HCoV-HKU1_N_06	HCoV-HKU1_02	HCoV-HKU1_03	HCoV-HKU1_08
HCoV-HKU1_N_08	HCoV-HKU1_02	HCoV-HKU1_07	HCoV-HKU1_08
HCoV-NL63_N_01	HCoV-NL63_01	HCoV-NL63_02	HCoV-NL63_03
HCoV-NL63_N_02	HCoV-NL63_01	HCoV-NL63_02	HCoV-NL63_06
HCoV-NL63_N_04	HCoV-NL63_04	HCoV-NL63_05	HCoV-NL63_06
HCoV-OC43_N_01	HCoV-OC43_01	HCoV-OC43_02	HCoV-OC43_03
HCoV-OC43_N_02	HCoV-OC43_01	HCoV-OC43_02	HCoV-OC43_07
HCoV-OC43_N_04	HCoV-OC43_04	HCoV-OC43_06	HCoV-OC43_07
SARS-CoV-2_N_01	SARS-CoV-2_01	SARS-CoV-2_02	SARS-CoV-2_03
SARS-CoV-2_N_02	SARS-CoV-2_04	SARS-CoV-2_05	SARS-CoV-2_06
SARS-CoV-2_N_03	SARS-CoV-2_12	SARS-CoV-2_13	SARS-CoV-2_14
MERS-CoV_N_01	MERS-CoVS-CoV_01	MERS-CoVS-CoV_02	MERS-CoVS-CoV_03
MERS-CoV_N_02	MERS-CoVS-CoV_01	MERS-CoVS-CoV_02	MERS-CoVS-CoV_06
MERS-CoV_N_03	MERS-CoVS-CoV_01	MERS-CoVS-CoV_05	MERS-CoVS-CoV_06
MERS-CoV_N_04	MERS-CoVS-CoV_04	MERS-CoVS-CoV_05	MERS-CoVS-CoV_06

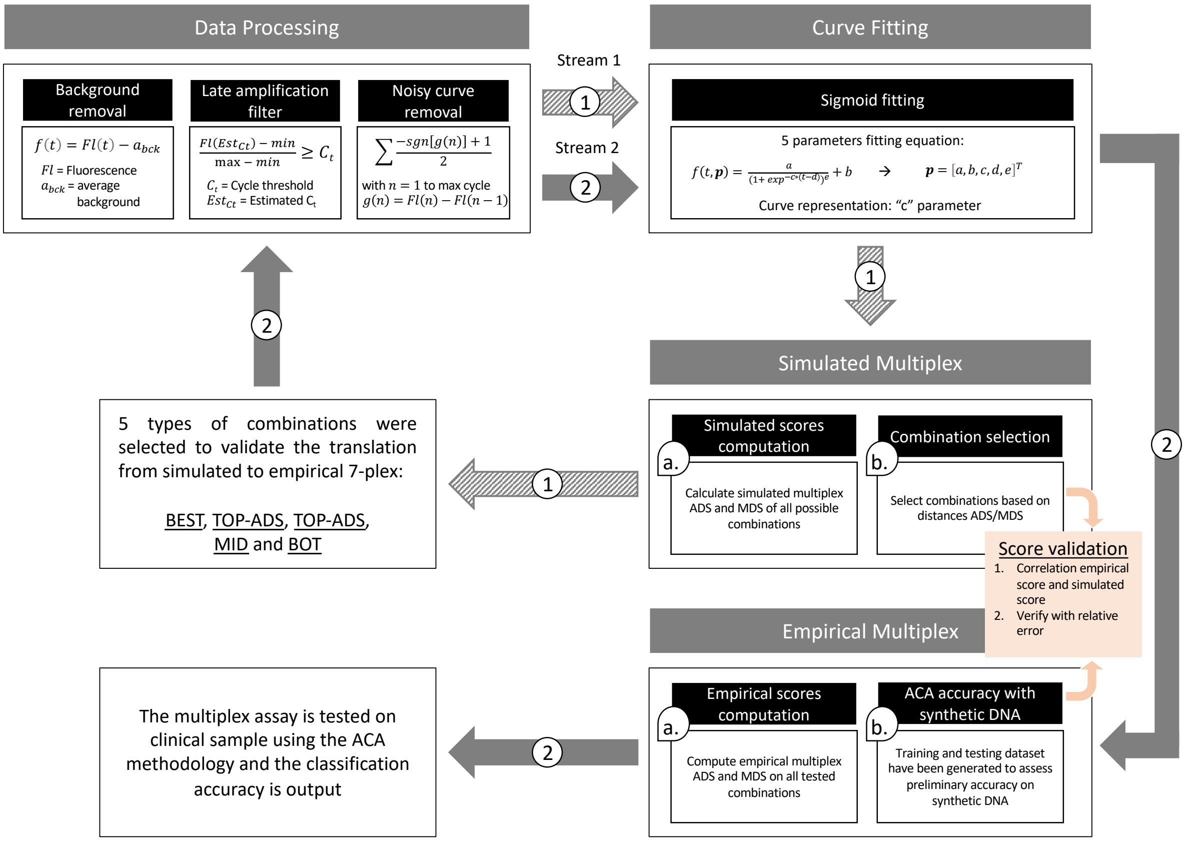

Supplementary Fig. 5 | Overall development of Smart-Plexer. Stream 1. Pipeline for combo selection based on simulated multiplex assays. Before combination selection, operations including 3-fold data manipulation (Background removal, Late amplification filter and Noisy curve removal), data processing (Sigmoid fitting and Curve FFI normalization) and simulated score computation (The types of data are raw curve, normalized curve, fitted parameters and "c" parameter) are conducted. The principle of selection is then based on MDS-ADS ranking system. Combinations from 5 groups (BEST, TOP-ADS, TOP-MDS, MID, BOT) are chosen for validation progress. **Stream 2**. Pipeline for result validation based on empirical multiplex assays. With empirical experiment, same pre-operations (3-fold data manipulation and data processing) are taken. Then, the empirical scores are computed, and the distributions of classification accuracy are evaluated versus scores. The last validation step is based on clinical samples with best assay combination developed so far.
