# Supplementary material for: Smart-Plexer: a breakthrough workflow for hybrid development of multiplex PCR assays
Source: Commun Biol. 2023 Sep 9;6:922. doi: 10.1038/s42003-023-05235-w (PMC10492832; doi:10.1038/s42003-023-05235-w)
Supplement: Supplementary file 3 — Description of Additional Supplementary Files [file 42003_2023_5235_MOESM3_ESM.pdf]

Description of Additional Supplementary Files

File name: Supplementary Data
Description: The source data used to generate Fig. 2, 3, 4 and 5 plots.
